# Nutritional and Technological Benefits of Pine Nut Oil Emulsion Gel in Processed Meat Products

**DOI:** 10.3390/foods14152553

**Published:** 2025-07-22

**Authors:** Berik Idyryshev, Almagul Nurgazezova, Zhanna Assirzhanova, Assiya Utegenova, Shyngys Amirkhanov, Madina Jumazhanova, Assemgul Baikadamova, Assel Dautova, Assem Spanova, Assel Serikova

**Affiliations:** 1 Department of Food Technologies, Shakarim University, 20A Glinki Str., 071412 Semey, Kazakhstan; b_1991@mail.ru (B.I.); zhanna.asirzhanova@yahoo.com (Z.A.); utegenova@yahoo.com (A.U.); shyngys_a@inbox.ru (S.A.); asemgul93@yandex.ru (A.B.); dautovaasel841@gmail.com (A.D.); asspanova78@gmail.com (A.S.); instantly@list.ru (A.S.); 2 Department of Bioengineering Systems, Shakarim University, 20A Glinki Str., 071412 Semey, Kazakhstan; madina.omarova.89@mail.ru

**Keywords:** emulsion gel, nutritional reformulation, pine nut oil, fatty acid profile, functional meat product

## Abstract

A high intake of saturated fats and cholesterol from processed meats is associated with increased cardiovascular disease risk. This study aimed to develop a nutritionally enhanced Bologna-type sausage by partially replacing the beef content with a structured emulsion gel (EG) formulated from pine nut oil, inulin, carrageenan, and whey protein concentrate. The objective was to improve its lipid quality and functional performance while maintaining product integrity and consumer acceptability. Three sausage formulations were prepared: a control and two variants with 7% and 10% EG, which substituted for the beef content. The emulsion gel was characterized regarding its physical and thermal stability. Sausages were evaluated for their proximate composition, fatty acid profile, cholesterol content, pH, cooking yield, water-holding capacity, emulsion stability, instrumental texture, microstructure (via SEM), oxidative stability (TBARSs), and sensory attributes. Data were analyzed using a one-way and two-way ANOVA with Duncan’s test (*p* < 0.05). The EG’s inclusion significantly reduced the total and saturated fat and cholesterol, while increasing protein and unsaturated fatty acids. The 10% EG sample achieved a PUFA/SFA ratio of 1.00 and an over 80% reduction in atherogenic and thrombogenic indices. Functional improvements were observed in emulsion stability, cooking yield, and water retention. Textural and visual characteristics remained within acceptable sensory thresholds. SEM images showed more homogenous matrix structures in the EG samples. TBARS values increased slightly over 18 days of refrigeration but remained below rancidity thresholds. This period was considered a pilot-scale evaluation of oxidative trends. Sensory testing confirmed that product acceptability was not negatively affected. The partial substitution of beef content with pine nut oil-based emulsion gel offers a clean-label strategy to enhance the nutritional quality of Bologna-type sausages while preserving functional and sensory performance. This approach may support the development of health-conscious processed meat products aligned with consumer and regulatory demands.

## 1. Introduction

Global dietary trends have increasingly emphasized the importance of healthier eating habits, driven by a heightened awareness of the relationship between food composition and chronic diseases such as cardiovascular ailments, obesity, and type 2 diabetes. In this context, consumers are demanding food products with improved nutritional profiles, particularly those with a reduced saturated fat content [1]. Bologna-type meat products, although widely consumed for their sensory appeal and convenience, are frequently criticized for their elevated levels of saturated fats and cholesterol—components closely linked to increased cardiovascular risk [2,3]. A substantial portion of these lipids originates from the beef component traditionally used in such formulations, which contributes both visible fat and intramuscular saturated fatty acids. Although fat plays a critical role in meat emulsions by enhancing mouthfeel, juiciness, and overall structure—largely due to its emulsifying properties and melt behavior during mastication [4]—reformulating products to lower consumers’ saturated fat intake remains a key objective in developing health-oriented alternatives.

In response to this challenge, researchers have explored various fat replacement strategies, including both lipid-based and non-lipid alternatives. Lipid-based strategies often employ unsaturated vegetable oils structured as oleogels, nanoemulsions, or emulsion gels to simulate the behavior of animal fats, while non-lipid strategies utilize hydrocolloids, dietary fibers, or protein fillers to replicate the functions of fats [2,3,5,6].

Among these, emulsion gels have shown particular promise due to their ability to mimic the structural and organoleptic characteristics of animal fats while offering a healthier lipid profile [7,8]. These gels are structured systems in which oil droplets are immobilized in a protein or polysaccharide gel matrix, enabling both functional mimicry and nutritional enhancement. They enable effective oil encapsulation, help moderate lipid oxidation under certain conditions, and preserve desirable rheological properties. The effectiveness of such gels, however, hinges on the selection of appropriate gelling agents and emulsifiers. In this study, the emulsion gel components were purposefully selected based on their known individual and synergistic contributions to nutritional and functional performance in meat matrices. Inulin serves as a prebiotic fiber with gel-forming properties and caloric reduction benefits [9,10], carrageenan contributes to thermal stability and water-binding capacity [11,12], and WPC serves both as an emulsifier and nutritional fortifier [13].

The fatty acid profile of beef muscle tissue is largely composed of saturated (SFAs) and monounsaturated fatty acids (MUFAs), with SFAs typically constituting around 40–50% of total lipids [14,15]. The main SFAs include palmitic (C16:0) and stearic acid (C18:0), with the latter being considered neutral regarding its impact on plasma cholesterol [16]. Oleic acid (C18:1n9c), a MUFA, often predominates among individual fatty acids in beef and is positively associated with improved flavor, oxidative stability, and health benefits [17]. Polyunsaturated fatty acids (PUFAs) such as linoleic (C18:2n6) and alpha-linolenic acid (C18:3n3) are present in lower concentrations, typically 2–7%, with grass-fed beef exhibiting higher n-3 PUFA levels than grain-fed cattle [18]. Given this limited PUFA content and the need for healthier formulations, this study aimed to partially replace the beef content with a structured emulsion gel to enhance the overall nutritional value of the sausages through an improved lipid profile and the inclusion of bioactive compounds [3,5,19].

In our recent work, we developed and characterized a novel emulsion gel (EG) composed of pine nut oil, inulin, carrageenan, and whey protein concentrate [19]. This gel is notably rich in unsaturated fatty acids, particularly oleic, linoleic, and alpha-linolenic acids, and contains bioactive components like tocopherols and phytosterols, known for their cardiovascular benefits [20]. While many emulsion gel formulations have used vegetable oils such as sunflower, canola, or olive oil, the use of pine nut oil distinguishes our formulation by contributing a unique profile of polyunsaturated fatty acids and natural antioxidants. Pine nut oil is particularly rich in linoleic and alpha-linolenic acids and offers a favorable omega-6 to omega-3 ratio, along with bioactive phytosterols and tocopherols that may promote lipid-lowering and anti-inflammatory effects. Furthermore, its mild, nutty flavor integrates well into meat matrices without compromising sensory attributes. These qualities support the use of pine nut oil as a functionally and nutritionally superior alternative to more commonly applied plant oils in emulsion gel systems [21,22]. By integrating this gel into the meat matrix, we not only reduce the intake of saturated fats derived from beef but also enrich the product with functional plant-based compounds. The reformulated Bologna sausages therefore meet the definition of functional foods, delivering health-promoting attributes beyond basic nutrition.

The primary objective of this study is to evaluate the feasibility and effectiveness of partially replacing the beef content with a structured emulsion gel in Bologna-type sausages. Specifically, it assesses the effects of this substitution on their physicochemical parameters, lipid profile, texture, and sensory quality. By targeting this underexplored application of pine nut oil-based emulsion gels, the study contributes to the development of nutritionally enhanced meat products that align with contemporary health trends and consumer expectations.

## 2. Materials and Methods

### 2.1. Materials

Spices were procured from the local marketplace in Semey and stored at 4 °C until further use. Grade I horsemeat, beef, and poultry meat (chicken) were sourced from certified local farms in Semey, Abay district. Prior to processing, muscle tissues were trimmed to remove excess fat and connective tissues and stored at 4 °C alongside the separated fat components. The emulsion gel (EG) was developed at the Department of Food Technologies, Shakarim University, Semey, Kazakhstan, and has been previously characterized in detail [19]. The EG composition included 55.23% moisture, 3.85% protein, 20.79% fat, and 1.28% ash. It was formulated using cold-pressed pine nut oil and exhibited a favorable fatty acid profile consisting of approximately 10.85% saturated fatty acids, 17.98% monounsaturated fatty acids, and 71.17% polyunsaturated fatty acids. The primary fatty acids were linoleic acid (C18:2, 70.19%), oleic acid (C18:1, 17.3%), and palmitic acid (C16:0, 6.81%). This composition supports the use of EG as a nutritionally beneficial and technologically functional alternative to animal-derived fat in emulsified sausage production. A detailed fatty acid profile is provided in Table S1 in the Supplementary Materials. All chemicals used in the study were of analytical grade.

### 2.2. Emulsion Gel Preparation

The EG was prepared by initially dispersing inulin in deionized water, which was at room temperature (~22–24 °C) and subsequently cooled to 18–20 °C, using high-speed homogenizer (IKA T25 digital Ultra-Turrax, IKA-Werke GmbH & Co. KG, Staufen, Germany) operating at 1000–1500 rpm for 10–15 min to ensure proper hydration and partial dissolution, followed by a 5 min rest for swelling. Carrageenan was then incorporated to enhance the gel’s strength. It was sprinkled into the inulin dispersion while it was stirred at the same speed for 5–7 min to prevent clumping and ensure uniform integration. Next, a lipid phase comprising pine nut oil and sunflower oil was gradually added to the hydrated inulin–carrageenan system, with homogenization speed increased to 1500 rpm for 15–18 min to form a homogeneous emulsion with uniformly dispersed oil droplets. The temperature was carefully kept below 30 °C throughout, using a refrigerated water bath (Julabo F12-ED, JULABO GmbH, Seelbach, Germany) to avoid premature gelation or emulsion destabilization. Once a visually homogeneous emulsion was achieved, whey protein concentrate (WPC) was introduced gradually over 1–2 min and mixed for another 2–3 min to ensure all protein was incorporated into the emulsion matrix. The final emulsion gel mixture was transferred to sterile containers and refrigerated at 0–4 °C for 6–8 h to allow for full gelation and network stabilization, after which it was evaluated for further use in food product formulations.

### 2.3. Bologna Sausage Formulation and Preparation

The sausages were formulated using a mixture of horsemeat, beef, and chicken, each characterized by specific proximate compositions. Horse meat (*M. semitendinosus*) (71.3% moisture, 21.02% protein, 6.30% fat, and 1.80% ash), first-grade beef (75.65% moisture, 21.16% protein, 2.15% fat, and 1.07% ash), and chicken (64.2% moisture, 21.64% protein, 11.45% fat, and 1.02% ash) were combined with modified starch and a standardized blend of curing agents and functional additives. The EG was incorporated as a functional reformulation component. All raw materials were ground using a meat grinder (TC22 Elegant Plus, Sirman, Italy) and mixed in a vacuum mixer (Mainca RM-20, Mainca, Barcelona, Spain) to ensure uniform distribution. Three experimental treatments were prepared: a control with no EG, and two treatment groups with the incorporation of 7% and 10% EG. The selection of 7% and 10% EG incorporation levels was based on preliminary trials aimed at identifying substitution ratios that could enhance nutritional quality while maintaining acceptable textural and sensory characteristics. The substitution of 7% and 10% EG was achieved by reducing the beef content to maintain overall formulation balance. This approach involves a deliberate reduction in the beef content, a major contributor to saturated fat and cholesterol in processed meats, by incorporating a structured emulsion gel. The goal was to improve the nutritional value of the product while retaining its processing characteristics and consumer-relevant sensory features. The full breakdown of each sausage formulation is shown in Table 1.

All meat components were inspected, trimmed, and cooled to 0–4 °C prior to processing. The meats were coarsely ground and mixed to ensure a uniform base. EG, starch, and the dry functional ingredients were incorporated during the fine chopping stage using a vacuum bowl cutter. Mixing was performed under temperature-controlled conditions, ensuring that the batter temperature remained below 12 °C to maintain protein functionality and emulsion stability.

Emulsification was carried out using a bowl cutter (Seydelmann K20, Maschinenfabrik Seydelmann KG, Stuttgart, Germany). The sausage batter was then stuffed into 120 mm diameter and 150 mm artificial casings (Amiflex T, JSC "LETPAKA",Šiauliai, Lithuania) using a vacuum filler (HP-25, Vemag Maschinenbau, Verden, Germany) and cooked in a smoking chamber at 78 °C until an internal temperature of 72 °C was achieved (35–40 min), which was monitored using a digital meat thermometer (Testo 105, Testo SE & Co. KGaA, Lenzkirch, Germany). The sausages were then rinsed with cold water and stored at 0–4 °C in a controlled-temperature refrigeration unit (Liebherr LGUex 1500, Liebherr Group, Bulle, Switzerland) for 18 days for subsequent physicochemical, textural, and sensory evaluations. The modified sausages were processed identically to the control group, with the only formulation change being the partial replacement of beef content with the structured emulsion gel.

**Figure 1:**
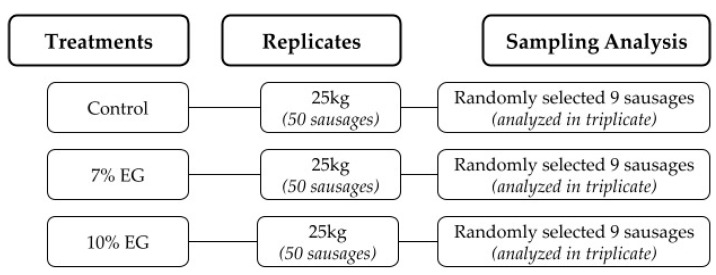
Sausage production and sampling workflow for analytical evaluation.

Each treatment group (control, 7% EG, and 10% EG) was produced with three independent replicates, with a production volume of 25 kg per replicate. From each replicate, approximately 50 sausages (∼500 g each) were produced. From every replicate, 9 sausages were randomly selected and divided into three sets of 3 sausages each to perform all analyses in triplicate (Figure 1).

### 2.4. Physicochemical Analysis

#### 2.4.1. Analysis of the Emulsion Gel System

The stability of the EG system was evaluated by subjecting it to centrifugal and thermal stress conditions. Centrifugal stability was assessed by centrifuging the emulsion at 330× g for 3 min using a benchtop centrifuge (Hettich Universal 320, Andreas Hettich GmbH & Co. KG, Tuttlingen, Germany), following the method described by Serdaroğlu et al. [23]. Creaming stability was determined after storing the samples at 4 °C for 7 days in a laboratory refrigerator (Liebherr LGUex 1500, Liebherr Group, Bulle, Switzerland) based on the protocol of the authors of [24]. Thermal stability was tested by heating the emulsion in a thermostatically controlled water bath (Memmert WNB 14, Memmert GmbH + Co. KG, Schwabach, Germany) at 70 °C for 30 min, as outlined in [25].

#### 2.4.2. Proximate Composition

The proximate composition of both the EG and the sausage samples was determined to evaluate their nutritional and functional profiles. For each sample, moisture, protein, fat, and ash contents were measured according to State Standard (ST) methods. Moisture content was determined by oven drying at 105 °C to constant weight (ST R 51479-99—Meat and meat products. Method for determining moisture content [26]), protein content was assessed using the Kjeldahl method with a nitrogen-to-protein conversion factor of 6.25 (ST 25011-81—Meat and meat products. Method for determining total nitrogen by the Kjeldahl method [27]), and fat content was quantified by Soxhlet extraction (ST 23042-86—Meat and meat products. Method for determining fat by Soxhlet extraction [28]). Ash content was measured by incineration in a muffle furnace at 550 °C (ST 31727-2012—Food products. Method for determining ash content [29]). State Standards officially adopted in Kazakhstan follow GOST regulatory guidelines commonly used across Central Asia and the CIS region. Public access to these standards is available through official portal such as https://www.gostinfo.ru. Carbohydrate content was calculated by difference. This analysis was performed on the EG alone and on all three sausage formulations (control, 7% EG, and 10% EG) to identify compositional shifts due to fat substitution. The energy value was calculated using conversion factors of 4 kcal/g for both carbohydrates and proteins and 9 kcal/g for fats.

The pH of both the EG and sausage samples was measured using a PHS-3D-03 calibrated digital pH meter (Shanghai San-Xin Instrumentation Inc., Shanghai, China) according to GOST R 51478-99108 [30]. Approximately 10 g of each sample was homogenized with 100 mL of distilled water, and the pH was recorded by immersing the electrode into the homogenate. Measurements were conducted in triplicate at room temperature to ensure accuracy and reproducibility. pH values were used to assess the impact of EG incorporation on product acidity and potential shelf stability.

#### 2.4.3. Color Measurement

Color measurements of the EG and sausage samples were performed using a calibrated colorimeter (Minolta CR-400, Konica Minolta, Japan) under D65 illumination and a 10° standard observer angle. The instrument was standardized using a white calibration tile prior to measurement. Color values were recorded in the CIE Lab* color space, in which L* indicates lightness (0 = black and 100 = white), a* represents the red–green axis, and b* corresponds to the yellow–blue axis. For EG, measurements were taken on the surface of the gel samples; for sausages, data were collected from the freshly cut cross-sectional surface of each sausage sample at three randomly selected points per sample. All measurements were conducted in triplicate, and results are expressed as mean values for statistical analysis. This approach enabled an objective comparison of color attributes across all formulations, providing insight into the visual effects of replacing beef content with EG.

### 2.5. Technological Characteristics of Sausage Batters

The water-holding capacity (WHC) of the sausage batters was assessed by subjecting the samples to heat and centrifugal treatment using a laboratory water bath (Memmert WNB 14, Memmert GmbH + Co. KG, Schwabach, Germany), followed by centrifugation in a benchtop centrifuge (Hettich Universal 320, Andreas Hettich GmbH & Co. KG, Tuttlingen, Germany). Emulsion stability (ES) was evaluated by quantifying the total expressible fluid (TEF) and expressible fat (EFAT) based on the procedure of Hughes et al. [31], using a precision analytical balance (Mettler Toledo ME204, Columbus, OH, USA). Jelly and fat separation (JFS) was expressed as a percentage of released liquid relative to the initial weight of the batter, as outlined in [32]. Processing yield (PY) was calculated as the percentage difference between the product weight before stuffing (W_1_) and after thermal processing (W_2_). These measurements provided insight into the functional performance of sausages formulated with emulsion gel, particularly in terms of cooking stability, juiciness, and oil-retention behavior.

### 2.6. Fatty Acid Profile and Cholesterol Determination

To assess the lipid content, total lipids were isolated from the sausage samples following the Folch method [33]. The extracted lipids were subsequently derivatized into fatty acid methyl esters (FAMEs) according to the Ce 2–66 protocol [34]. Analysis of FAMEs was performed using a gas chromatograph (HP 6890, Agilent Technologies, Santa Clara, CA, USA) equipped with a DB-23 fused-silica capillary column (60 m × 0.25 mm internal diameter and 0.25 μm film thickness). Both the injector and detector temperatures were set to 250 °C. The oven temperature program involved an initial isothermal step at 140 °C for 5 min, followed by an increase of 4 °C/min until reaching 240 °C, with a final hold for 10 min. Fatty acids were identified by comparing retention times with those of known standards.

For cholesterol analysis, an Agilent 1260 high-performance liquid chromatography (HPLC) system coupled with a diode array detector (DAD) was used (Agilent Technologies, Santa Clara, CA, USA). Detection was conducted at a wavelength of 210 nm. Cholesterol quantification was carried out after direct saponification, following procedures described in previous studies [35,36]. Chromatographic separation was achieved on a Spherisorb S5ODS2 column (250 mm × 4.6 mm, 5 µm particle size; Waters Corporation, Milford, MA, USA), maintained at 25 °C. The mobile phase consisted of a 70:30 mixture of methanol and isopropanol (*v*/*v*), and the flow rate was set to 1 mL/min.

### 2.7. Microstructural Analysis

The internal architecture of the sausage formulations was examined using scanning electron microscopy (SEM) to evaluate the structural modifications introduced by emulsion gel incorporation. Prior to imaging, the samples were freeze-dried to retain their native morphology. After drying, specimens were mounted on aluminum holders and coated with a thin gold layer using a sputter coater to enhance surface conductivity. SEM analysis was performed using a JEOL JSM-IT100 scanning electron microscope (JEOL Ltd., Tokyo, Japan) at varying magnifications. The resulting micrographs enabled observation of the protein network, the dispersion and integration of fat and gel particles, and the occurrence of voids or fibrous structures. Comparative analysis between the control and EG-treated sausages provided insights into how the gel system impacted structural integrity, matrix network development, and component distribution within the sausage microenvironment.

### 2.8. Textural Analysis

To determine the mechanical characteristics of the sausages, including parameters such as hardness, springiness, cohesiveness, chewiness, and resilience, a texture analyzer (TA.XTplus, Stable Micro Systems Ltd., Surrey, UK) equipped with a cylindrical probe (35 mm diameter) was employed. Uniform sausage portions (20 mm height × 20 mm diameter) were compressed to 50% of their initial height through two consecutive compression cycles at a constant speed of 1.0 mm/s, with a 5 s interval between compressions. Hardness was identified as the peak force during the first cycle, while springiness reflected the ability of the sample to regain its shape post-compression. Cohesiveness was determined as the ratio of the area under the second compression curve to that of the first. Chewiness was calculated by multiplying hardness, cohesiveness, and springiness. Resilience was assessed as the material’s capability to recover during decompression. All analyses were conducted at ambient temperature in triplicate to assess the effects of emulsion gel addition on the textural attributes of the control and treated sausages.

### 2.9. Oxidative Stability

The extent of secondary oxidation in the meat samples was determined by measuring the concentration of thiobarbituric acid reactive substances (TBARSs), following the method described by [37]. Absorbance was recorded at 532 nm using a UV–Vis spectrophotometer (UV-1800, Shimadzu Corp., Kyoto, Japan). This method quantifies lipid peroxidation products and expresses the results as milligrams of malondialdehyde (MDA) per kilogram of sample. All chemicals used, including 2-thiobarbituric acid and trichloroacetic acid, were of analytical grade (Sigma-Aldrich, St. Louis, MO, USA).

### 2.10. Sensory Evaluation

Sensory evaluation of the sausage samples was carried out to assess consumer-relevant quality attributes influenced by the incorporation of EG. A panel of 12 untrained but regular sausage consumers participated in the evaluation. Prior to the test, the panelists were briefed on the evaluation procedure and the use of a 9-point hedonic scale, on which 1 indicated “dislike extremely” and 9 represented “like extremely.” Each panelist was provided with coded samples representing the control, 7% emulsion gel, and 10% emulsion gel formulations. The attributes evaluated included appearance, aroma, flavor, texture, and overall acceptability. Sausage samples were cut into uniform slices, served at room temperature under standardized lighting conditions, and presented in randomized order to minimize bias. Water and saltless bread were provided between samples to cleanse the palate [38]. The use of untrained panelists in such preliminary product development is consistent with established practices in meat product reformulation, in which early-stage sensory testing aims to assess perceptible differences prior to broader consumer trials [39].

### 2.11. Statistical Analysis

All statistical evaluations were conducted using SPSS software (version 21.0; IBM Corp., Armonk, NY, USA). To determine the influence of sausage formulations on parameters such as proximate composition, fatty acid composition, cholesterol levels, functional characteristics, texture profile, color measurements, and sensory evaluation, one-way analysis of variance (ANOVA) was employed. When significant variation was detected (*p* < 0.05), Duncan’s multiple range test was used to differentiate between group means. For lipid oxidation (measured via TBARSs), a two-way ANOVA was applied to examine the interaction effects between formulation type and storage duration. Post hoc comparisons were further performed using the least significant difference (LSD) method at the 5% significance threshold. All assessments were conducted in triplicate across three independent processing replicates, and results are presented as mean ± standard error of the mean.

## 3. Results

### 3.1. Analysis of the Emulsion Gel System (Color, pH, and Thermal Stability)

The EG’s color attributes, pH, and thermal stability were assessed immediately after production and again after 7 days of refrigerated storage at 4 °C. The evaluated parameters included lightness (L*), redness (a*), yellowness (b*), pH, and total fluid release, as presented in Table 2.

### 3.2. Proximate Composition and Energy Values

Table 3 presents the proximate composition and caloric values for both the control sausages and those reformulated with 7% and 10% EG. Substituting the beef content with the EG led to statistically significant changes (*p* < 0.05) in moisture, protein, and fat contents, depending on the substitution level.

### 3.3. Technological Characteristics of the Sausage Batter

The technological characteristics of the sausage batter, including its water-holding capacity (WHC), emulsion stability (ES), jelly and fat separation (JFS), and processing yield (PY), are critical indicators of the functional performance of fat-reduced meat products. Table 4 presents the results obtained for the control, 7% EG, and 10% EG formulations.

### 3.4. Fatty Acid Profile, Total Cholesterol, and Nutritional Facts of the Sausages

As consumer demand for healthier foods rises, efforts to reduce saturated fats and improve lipid profiles in meat products have intensified. Table 5 shows the fatty acid composition, cholesterol content, and nutritional indices of all sausage formulations.

### 3.5. Microstructural Analysis Results

**Figure 2:**
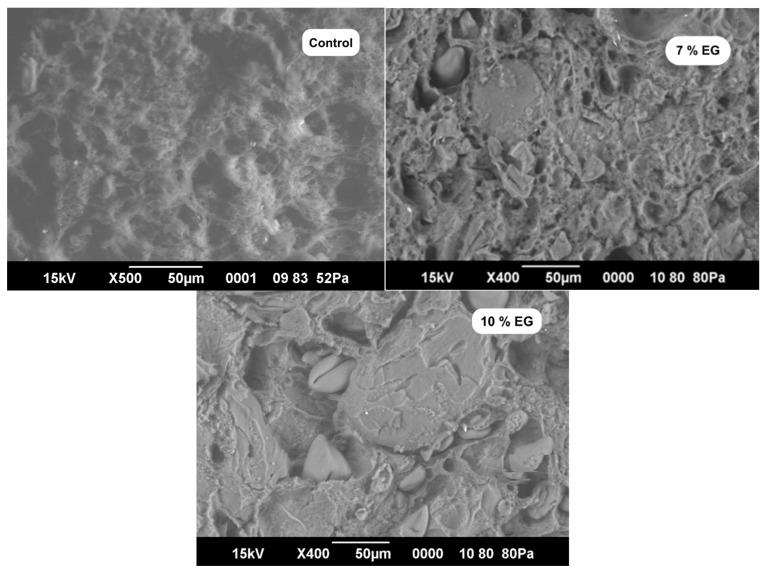
Scanning electron micrographs of sausage samples showing microstructural changes associated with control, 7% EG, and 10% EG formulations.

Figure 2 illustrates the SEM micrographs of the sausage samples, highlighting the microstructural alterations induced by the incorporation of EG.

### 3.6. Color and Texture of the Sausages

Table 6 shows how the incorporation of EG significantly influenced the color and texture attributes of the sausages.

### 3.7. Oxidative Stability Results

**Figure 3:**
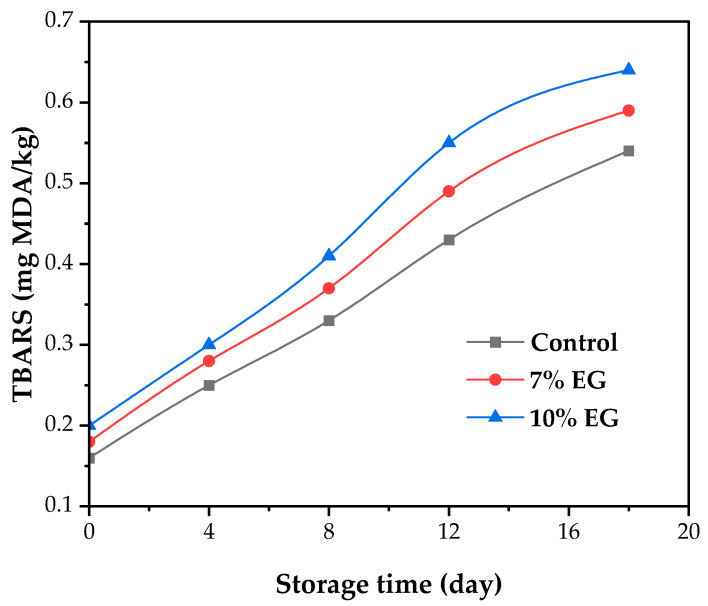
TBARS (mg MDA/kg) values of control and reformulated Bologna sausages at 0, 4, 8, 12, and 18 days of storage at 4 °C.

Lipid oxidation represents a significant challenge in the reformulation of meat products, especially when animal fats are substituted with unsaturated oils. Due to the high degree of unsaturation in the oil blend used in the EG formulations, there is an increased likelihood of oxidative degradation during storage. Figure 3 depicts the trend of TBARS values in both the control and reformulated sausage samples over 18 days of refrigerated storage.

### 3.8. Sensory Evaluation Results

Sensory evaluation is a critical aspect of assessing the consumer acceptance of reformulated meat products. As presented in Table 7, the incorporation of EG at both 7% and 10% levels resulted in acceptable sensory attributes without significant deviations from the control (*p* < 0.05).

## 4. Discussion

The EG displayed a characteristic light-yellow hue upon production, with a b* value of 20.95, indicative of mild yellowness that is suitable for influencing the appearance of reformulated meat products such as bologna (Table 2). Over 7 days of storage, the gel’s lightness (L*) increased, while the redness (a*) and yellowness (b*) values significantly decreased, indicating visible pigment fading. These changes are attributed to color instability related to mild oxidative or structural shifts in the gel matrix during cold storage. Similar trends were documented by Pintado et al. [40], who observed increases in lightness and decreases in redness in meat products formulated with oil-based emulsion gels, linking the shifts to structural reorganizations and pigment instability during cold storage. Alejandre et al. [41] also found that carrageenan-structured gels with algae oil showed reduced a* and b* values due to carotenoid oxidation and matrix interactions over time. From a formulation standpoint, the observed alterations in color may influence how consumers judge the freshness and overall quality of the product. While the increase in lightness is noticeable, the decline in redness and yellowness could create a visual mismatch compared to traditional sausage products. This underlines the importance of selecting appropriate oils and natural pigments when designing EG systems to ensure a consistent appearance. Nevertheless, the degree of change stayed within acceptable sensory boundaries, and the lighter appearance might even be interpreted by consumers as indicative of a lower-fat or healthier alternative.

The pH of the EG was initially measured at 5.28, a value primarily determined by the mildly acidic buffering effects of whey protein concentrate (WPC) and carrageenan. WPC contributes to acidic functional groups, while carrageenan provides sulfate esters that promote gelation and slightly reduce the system’s pH. In contrast, inulin is a neutral polysaccharide, and the oils (pine nut and sunflower) do not significantly influence pH. After 7 days of storage, the pH increased slightly but significantly to 5.45, likely due to the mild matrix reconfiguration and protein interactions. A stable pH within this narrow range is beneficial as it maintains the electrostatic environment required for proper protein–protein interaction in meat emulsions. In the study by Pintado et al. [40] on chia and oat EGs used as animal fat replacers in fresh sausages, the authors reported that the pH of their emulsion gel systems remained stable during refrigerated storage. They noted that no significant pH differences were detected between the fresh and stored samples, reflecting their good buffering capacity and structural consistency when used in meat batters. A comparable pH stability was documented by Serdaroğlu et al. [42] in gels containing inulin and proteins, indicating predictable gel behavior during processing. The modest rise in pH observed during storage may reflect gradual adjustments in the internal structure of the emulsion gel, potentially linked to protein unfolding or reorganization at the oil–water interface. Given that pH significantly influences the charge dynamics and binding capacity of gelling agents and emulsifiers, its stability within a narrow margin suggests that the emulsion gel is unlikely to interfere with protein–protein interactions in the meat matrix. This pH consistency supports reliable thermal performance and helps maintain microbial safety during storage. Similar observations on the functional impact of pH in biopolymer-stabilized emulsions have been reported by Dickinson [43], highlighting its importance in maintaining emulsion structure and compatibility in food systems.

The emulsion gel formulation exhibited high physical stability when subjected to centrifugation, with no visible phase separation observed under this condition. Regarding creaming stability, the emulsion gel retained a stability of over 90% without noticeable turbidity after being stored for 7 days at 4 °C, suggesting strong resistance to separation. Comparable stability behavior has been associated with inulin-containing emulsion gels, as reported in prior studies in which inulin contributed to phase stability and oil entrapment under thermal and centrifugal stress conditions [42,44].

Thermal stability is a critical attribute for EGs intended for heat-processed meat products. The gel formulated in this study demonstrated excellent structural resilience when exposed to thermal and storage-related stress. This was supported by the minimal fluid release observed, measured at just 1.06% immediately after processing and 1.38% following 7 days of refrigeration (Table 2). No syneresis or visible exudation was observed during storage or handling. The minimal fluid release reflects a strong gel matrix and efficient water–oil binding, which are critical for maintaining cooking yield and preventing textural defects in sausages. A gel with poor thermal stability would release fat and moisture during cooking, leading to product shrinkage and reduced sensory appeal. The low fluid loss confirms that the gel is highly compatible with thermal processing conditions, making it suitable for commercial sausage manufacturing. Furthermore, their high structural stability enhances the reproducibility and handling convenience of EGs during industrial-scale processing.

Previous studies have indicated that proteins such as soy protein isolate, meat protein, or sodium caseinate, often combined with enzymes like transglutaminase, can enhance gel firmness and reduce fluid separation in emulsified systems [45,46,47]. In contrast, our formulation achieved high structural integrity without relying on enzymatic crosslinking agents. The combination of whey protein concentrate (WPC) and carrageenan provided sufficient network formation and elasticity, enabling the EG to retain water and fat effectively during thermal processing. This strategy aligns with the clean-label trend, offering a formulation that avoids enzyme additives and appeals to consumers seeking simpler, minimally processed food products. The successful use of only food-grade biopolymers demonstrates both the scalability and regulatory feasibility of our approach, particularly in markets in which processing aids like transglutaminase are restricted. Previous research supports the feasibility and effectiveness of our clean-label EG formulation. Sato et al. [45] demonstrated that emulsified gels developed using protein–polysaccharide systems exhibited enhanced oxidative and pH stabilities, emphasizing the structural benefits of biopolymer interactions. Pintado et al. [46] reported that oil-in-water EGs stabilized with chia and cold gelling agents achieved desirable technological properties without requiring enzymatic crosslinking. Similarly, Herrero et al. [47] confirmed the structural integrity and oil-binding efficiency of polysaccharide-based gels functioning as fat mimetics. These studies collectively substantiate the design of our formulation, which achieves strong matrix stability and thermal resistance using only food-grade ingredients, without reliance on enzymatic stabilizers.

Although the fat content was progressively reduced in the reformulated sausages, all samples maintained acceptable technological functionality and sensory quality. The decrease in fat content led to a statistically significant reduction in energy values (*p* < 0.05), with the total caloric content dropping from 180.61 kcal/100 g in the control to 164.85 kcal/100 g in the 10% EG formulation (Table 3). Similarly, energy derived from fat decreased significantly, from 109.89 kcal in the control to 95.13 kcal in the 10% EG sample (*p* < 0.05), in line with the lower lipid concentration. In contrast, Jiménez-Colmenero et al. [25] reported higher caloric values of 225–245 kcal/100 g for control and reformulated frankfurters, with fat contributing approximately 70% of the total energy. In our study, energy contributions from fat were notably lower across all formulations, with the proportion of energy from fat decreasing from 60.8% in the control to 57.7% in the 10% EG group. This further illustrates the nutritional benefits of incorporating the emulsion gel matrix. Furthermore, the ash content increased slightly but significantly in the 10% EG formulation (*p* < 0.05), likely reflecting the mineral-rich composition of the added gel components. These results are consistent with earlier findings by de Souza Paglarini et al. [48] and Ashakayeva et al. [49], who described enhanced protein levels and reduced fat content in sausages formulated with protein- and fiber-based EGs. Similar outcomes were observed by Oppong et al. [50], supporting the ability of EG systems to improve water retention and adjust nutritional composition. The pH values of the sausage batters decreased significantly with increasing levels of EG incorporation, ranging from 6.12 in the control to 5.43 in the 10% EG sample. This drop in pH is attributed to the inherent acidity of the EG components, particularly inulin and whey protein concentrate, which have buffering effects and slightly acidic profiles. Such pH modulation may influence protein solubility and water-holding capacity, potentially enhancing the emulsion stability and cooking yield of the final product. According to European Parliament [51] labeling criteria, a fat reduction exceeding 30% qualifies a product as “fat-reduced.” While the 10% EG formulation achieved a fat reduction of approximately 13.4% compared to the control, this falls below the 30% threshold required for “reduced-fat” labeling under EU regulation [51]. Nonetheless, these findings highlight potential for further formulation optimization to enhance fat reduction while maintaining product quality.

Fat reduction typically poses risks to the stability and processing behavior of meat systems due to increased moisture and decreased lipid emulsification potential. However, the addition of structured emulsion gel counteracted these drawbacks by enhancing the WHC and reducing both the TEF and EFAT (*p* < 0.05) (Table 4). The 10% EG sample achieved the highest WHC (89.8%) and the lowest levels of expressible fluid and fat, suggesting robust gel matrix formation and effective moisture entrapment. These improvements may be attributed to the hydrophilic and gel-strengthening components of the EG, namely carrageenan, WPC, and inulin—which effectively interact with the meat protein network to improve water binding.

The JFS values also decreased significantly (*p* < 0.05) with higher EG incorporation (Table 4), indicating a reduction in phase separation and an improvement in fat retention properties. Similar improvements were observed in earlier studies using protein-stabilized EG systems [52,53]. In particular, Urgu-Öztürk et al. [52] demonstrated that using hazelnut-based pre-emulsion systems in beef sausages significantly improved emulsion stability and water retention, while Nieto and Lorenzo [53] reviewed multiple applications of olive oil as a fat replacer and reported that appropriate emulsion strategies could help maintain textural and technological quality in low-fat meat products. The processing yield (PY) improved linearly with the increased EG content, reaching 96.5% in the 10% EG treatment (*p* < 0.05). This trend correlated positively with the WHC (r = 0.836, *p* < 0.01) and negatively with both the TEF (r = −0.842, *p* < 0.01) and JFS (r = −0.874, *p* < 0.01), indicating the stabilizing effect of the EG system during thermal processing. The enhanced technological functionality may also stem from the emulsion’s ability to trap water and fat within its microstructure during chopping and cooking, contributing to greater retention and less exudation. Similar findings have been reported by the authors of [54,55], who showed the combination of functional binders and emulsifiers helped maintain emulsion stability in reduced-fat formulations. Specifically, Aktaş and Gençcelep [54] showed that using modified starches in sausages could improve emulsion stability and moisture control, and Alejandre et al. [55] found that replacing animal fat with canola oil-based hydrogels and organogels led to reduced fat loss and improved cooking yield in meat batters. Overall, the results demonstrate that structured EGs can significantly improve the technological performance of sausage batters subjected to fat reduction, ensuring product yield and integrity while supporting fat and calorie reduction strategies.

Substituting beef fat with the pine nut oil-based EG significantly decreased SFAs from 56.95% in the control to 36.69% and 20.75% in the 7% and 10% EG samples, respectively (*p* < 0.05), reflecting the high unsaturation of the lipid blend (Table 5). Concurrently, MUFAs, predominantly oleic acid, rose from 30.20% to 45.14%, while PUFAs—the sum of linoleic and linolenic acids—increased from 4.35% in the control to 26.79% in the 10% EG group (*p* < 0.05). These trends align with previous findings from formulations using peanut–linseed oil gels [44] and linseed-based gelled emulsions [56], both of which demonstrated similar improvements in lipid composition after the partial substitution of animal fat with unsaturated oil matrices.

Cholesterol levels showed a significant decline, dropping from 62.3 mg/100 g in the control to 43.7 and 28.4 mg/100 g in the 7% and 10% EG groups, respectively (*p* < 0.05), indicating a reduction of approximately 54% in the group with the highest substitution level. This outcome corresponds with observations reported by Glisic et al. [57], who also documented cholesterol reduction through linseed oil gel substitution in dry fermented sausages, and supports broader conclusions from other structured oil-based fat replacement systems [44].

The PUFA/SFA ratio, a key index of lipid quality, increased from 0.08 in the control to 1.29 in the 10% EG sample, exceeding the FAO/WHO minimum recommendation of 0.45. Furthermore, the n-6/n-3 ratio improved from non-detectable in the control to 3.72, which is within the optimal dietary range. Cardiovascular risk indicators—the index of atherogenicity (IA) and index of thrombogenicity (IT)—were significantly reduced from 1.14 and 3.30 in the control to 0.20 and 0.41, respectively, in the 10% EG sausages (*p* < 0.05), indicating a favorable shift toward a cardio-protective lipid profile. These improvements are consistent with previous studies on EGs made from chia mucilage–olive oil [58] and olive–walnut–chia blends [59], which also reported enhanced PUFA/SFA ratios, decreased cholesterol, and lowered IA/IT indices while preserving product quality. Furthermore, Franco et al. [60] demonstrated that partially replacing pork backfat with linseed oil-based oleogels in fermented sausages significantly enhanced the PUFA/SFA ratio while preserving product quality. Similarly, Pintado and Cofrades [61] showed that replacing animal fat with emulsions or oleogels structured from olive and chia oils improved the nutritional value and maintained acceptable sensory and technological characteristics in dry fermented sausages.

The nutritional improvements observed in the reformulated sausages align with the European Union’s regulatory framework on nutrition claims. According to Regulation (EC) No 1924/2006, products containing at least 0.6 g of alpha-linolenic acid (ALA) per 100 g may be labeled as “high in omega-3 fatty acids” [51]. In this study, the 10% EG formulation exceeded this threshold, supporting its eligibility for such a claim. Furthermore, the reduction in total and saturated fats—alongside improved PUFA/SFA ratios—positions the product within the parameters of health-oriented dietary guidance, including recommendations from the European Food Safety Authority (EFSA) [62] and the FAO/WHO [63], both of which advocate reducing saturated fat intake while increasing unsaturated fat consumption to mitigate cardiovascular risk. The integration of these nutritional features suggests that sausages formulated with pine nut oil-based EG can not only meet functional food criteria but also comply with regulatory standards for nutrition and health claims, enhancing their potential marketability and public health impact.

The SEM micrographs presented in Figure 2 reveal significant differences in the internal structure of the sausage samples depending on the level of EG incorporation. The control sample (without EG) exhibited a porous, less cohesive matrix with visibly larger voids, indicative of less efficient fat and water entrapment within the protein network. In contrast, both the 7% and 10% EG formulations displayed more compact and denser microstructures, characterized by uniformly distributed lipid droplets and fewer gaps. This morphological shift suggests a reinforced protein–polysaccharide matrix, likely due to the stabilizing effects of carrageenan, whey protein concentrate, and inulin present in the EG. Similar structural improvements have been reported by Nacak et al. [44], who observed increased matrix uniformity and reduced interstitial voids in sausages containing peanut and linseed oil emulsion gels. The denser network observed in the 10% EG sample further supports its superior technological performance, as shown by its reduced amount of expressible fluid, higher water-holding capacity, and improved processing yield. These findings collectively indicate that EG inclusion reinforces the gel–protein interaction during processing, leading to more stable and functionally efficient sausage matrices.

Color is a key quality attribute in meat products, as visual changes can strongly influence consumer acceptance [64]. According to Table 6, the addition of EG significantly affected the sausage color parameters. Both the 7% and 10% EG formulations exhibited increased lightness (L*) values (67.30 and 67.85, respectively) compared to the control (64.80), likely due to elevated moisture and the intrinsic whitening effect of inulin and whey protein in the EG matrix. A gradual reduction in redness (a*) was noted with increasing EG inclusion (from 14.85 to 13.10 and 13.15), possibly due to the dilution of meat pigments like myoglobin. Simultaneously, yellowness (b*) values rose from 11.80 in the control to 14.10 in the 10% EG sample, reflecting the presence of carotenoids and the redistribution of the lipid phase. These patterns are consistent with earlier studies on fat-reduced meat systems using biopolymer-stabilized emulsions. Ashakayeva et al. [49] noted that pumpkin-based emulsion gels elevated lightness and yellowness in semi-smoked horsemeat sausages due to the pigments and fiber content in the gel. Similarly, Alejandre et al. [55] reported increased L* and b* values in meat batters formulated with canola oil-based hydrogels, linking the change to lipid dispersion and matrix hydration. Huang et al. [64] also demonstrated that konjac glucomannan and soy protein isolate gels improved lightness and maintained product consistency in fat-reduced models. In another study, Pintado and Delgado-Pando [65] emphasized the visual impact of plant-derived extenders on meat formulations, while Alejandre et al. [66] showed that microalgal oil gels enriched with natural antioxidants enhanced yellowness and oxidative stability in reduced-fat patties. These findings reinforce that color shifts in our EG-based sausages reflect not only the functional behavior of added ingredients, but also broader trends observed in similar reformulation strategies.

A texture profile analysis (TPA), as summarized in Table 6, revealed that the inclusion of emulsion gel (EG) induced notable structural changes in the sausage matrix. The hardness increased significantly in both the 7% and 10% EG formulations (9.65 N and 9.35 N, respectively) compared to the control (8.00 N), suggesting a firmer matrix likely resulting from the interactions between carrageenan and whey proteins. These outcomes are consistent with those of Youssef and Barbut [67], who reported that higher protein levels and specific fat/oil emulsions can enhance emulsion stability and texture through better protein–fat interactions. The authors of [59] also observed improved firmness in bologna-type sausages when animal fat was completely replaced with oil-based EGs stabilized with chitosan, indicating the structural benefits of such systems in processed meat products. The observed firmness may also relate to an increased moisture and protein content, contributing to a denser matrix structure, as supported by Freire et al. [68], who demonstrated that gelled double emulsions could improve the functional texture of pork patties by retaining structural moisture. Conversely, cohesiveness and chewiness decreased with rising EG levels (from 0.54 to 0.35 and 3.70 to 2.75, respectively), likely due to the reduced fat content and altered interactions among fat, protein, and water during thermal processing. Similar findings were noted by the authors of [69], who used inulin-based emulsion-filled gels in fermented sausages and found that fat reduction disrupted typical fat–protein interactions, influencing cohesiveness. Springiness remained largely unchanged across treatments, suggesting that the elastic recovery of the samples was preserved. These textural trends align with those in the study by Domínguez et al. [70], who emphasized the role of gel-based systems in maintaining structural integrity while optimizing lipid profiles, and Fu et al. [71], who systematically evaluated various oleogels as fat replacers, showing that textural parameters such as springiness could be maintained when gels are properly formulated. Overall, the data confirm that EG-based reformulations can enhance or maintain key textural characteristics while supporting fat reduction strategies in meat product development.

Vegetable oils, particularly those rich in polyunsaturated fatty acids, may increase susceptibility to lipid oxidation, depending on their fatty acid profile and the surrounding matrix. The high degree of unsaturation in the oils used for the EG formulations enhances the risk of oxidative degradation during storage. As shown in Figure 3, TBARS values rose progressively over 18 days of refrigerated storage in all samples, indicating increased lipid peroxidation. This duration was designed as a pilot-scale evaluation to explore short-term oxidative trends rather than to define their commercial shelf-life. At day 0, TBARS values were low in all treatments, starting at approximately 0.164 mg MDA/kg in the control, and these were slightly higher in the 7% EG (0.183 mg MDA/kg) and 10% EG (0.191 mg MDA/kg) samples. During storage, oxidation markers increased significantly (*p* < 0.05), with the 10% EG group showing the highest value at day 18 (0.654 mg MDA/kg), compared to 0.587 mg MDA/kg for the 7% EG and 0.541 mg MDA/kg for the control. Further studies are needed to validate the long-term stability and safety of these formulations under commercial storage conditions. Future work should include microbial analyses, spoilage kinetics, and extended storage evaluations (e.g., 30–60 days) to better reflect real-world shelf-life requirements.

The elevated TBARS levels in the EG groups can be attributed to the use of unsaturated vegetable oils, which are more reactive to oxidation. The formulation’s composition, particularly the use of omega-3 rich oils like chia or walnut, may explain this heightened sensitivity. While enzymatic agents such as transglutaminase are often associated with structural improvements in gel matrices, they may also influence oxidation dynamics via protein–lipid interactions. Nevertheless, all TBARS values remained well below the sensory detection threshold of 1.36 mg MDA/kg for rancidity, suggesting that the lipid stability of the products was maintained throughout storage.

The inclusion of pine nut and sunflower oils in the EG appears to have contributed to limiting oxidative changes during storage. These oils are known to contain natural antioxidants—pine nut oil offers polyphenols and tocopherols, while sunflower oil is particularly rich in α-tocopherol. Such compounds are capable of neutralizing free radicals and reducing the formation of lipid peroxides, even in systems with elevated levels of unsaturated fats. The structure of the EG may also have provided oxidative protection by entrapping lipid droplets within the matrix, thereby limiting their exposure to oxygen, as previously demonstrated by the authors of [67], who reported that well-emulsified meat batters with a higher protein content formed more stable matrices that minimized lipid dispersion and oxidative degradation. Similarly, Domínguez et al. [70] highlighted that novel gelation systems, including emulsion gels, can encapsulate unsaturated fats within a structured network, thereby improving lipid stability and delaying oxidation in reformulated meat products. Although TBARS values were moderately higher in the reformulated samples, they remained below sensory detection thresholds, indicating that their oxidative quality was effectively preserved. These findings support the role of oil selection and gel structure in maintaining the stability of fat-reduced sausage products.

Future research should focus on optimizing antioxidant strategies tailored to EG systems, including the targeted incorporation of natural plant-derived phenolics (e.g., rosemary, grape seed, and green tea extracts) or synergistic combinations of lipid- and water-soluble antioxidants. Furthermore, exploring microencapsulation techniques or nanoemulsified antioxidant carriers could further enhance oxidative stability without altering the clean-label character of the formulation.

Sensory evaluation is a critical aspect of assessing consumer acceptance of reformulated meat products. As presented in Table 7, the incorporation of EG at both the 7% and 10% levels resulted in acceptable sensory attributes without significant deviations from the control (*p* < 0.05). The panelists noted that all formulations maintained desirable characteristics in appearance, flavor, and texture. Interestingly, the color attributes of sausages containing the EG closely resembled that of the control group, with no perceptible differences that negatively influenced consumer perceptions. This indicates that the use of pine nut oil-based EG did not significantly alter the visual quality of the final product, likely due to the balanced formulation and the neutral pigment profile of the gel matrix. This is in line with what was previously reported by Pintado et al. [65] and Alejandre et al. [56], who reported that well-formulated emulsion gels can preserve the typical coloration of meat products even at higher substitution levels. Texture and flavor were also positively rated, particularly in the 10% EG sample, which scored marginally higher than the 7% sample. This suggests that the structural integrity and organoleptic properties were effectively maintained or enhanced due to the gel’s contribution to moisture retention and mouthfeel. Franco et al. [60] observed that linseed oil oleogels maintained the structural and textural integrity of fermented sausages, while Pintado and Cofrades [61] reported comparable outcomes using chia–olive oil gels, demonstrating strong stability and high consumer acceptability. Overall, the results demonstrate that pine nut oil-based EG can serve as a viable fat replacer in emulsified sausages without compromising key sensory characteristics.

While inulin was selected for its functional and nutritional attributes, it is important to note that this dietary fiber may trigger gastrointestinal discomfort or allergic responses in individuals with conditions such as irritable bowel syndrome (IBS) or inflammatory bowel disease (IBD). Future work should explore alternative dietary fibers such as citrus fiber, oat β-glucan, or psyllium husk, which may offer similar structural and prebiotic benefits with a lower risk of intolerance for sensitive consumer groups.

## 5. Conclusions

This study confirms the feasibility of using a pine nut oil-based EG as a partial replacer of beef content in Bologna-type sausages to improve nutritional quality. The substitution resulted in lower levels of saturated fat and cholesterol while enhancing the unsaturated fatty acid profile, without compromising sensory acceptance or textural integrity.

The clean-label emulsion system maintained functional performance and provided moderate oxidative protection during short-term storage. As this was a pilot-scale evaluation, future studies should include extended oxidative monitoring and microbial stability assessments to support shelf-life validation and commercial application.

## 6. Limitations and Future Work

This study was limited to a short-term, 18-day refrigerated storage period, which does not fully reflect the commercial shelf-life expectations for processed meat products. While the oxidative stability remained within acceptable sensory thresholds, the microbial stability was not assessed, and no accelerated storage or distribution simulations were conducted. Additionally, the sensory evaluation was performed with a small, untrained panel, which may not capture broader consumer preferences. Future research should involve extended storage trials, including microbiological and spoilage analyses, to validate product safety and shelf-life under real-world conditions. Expanding sensory testing to larger and demographically diverse consumer groups, as well as exploring alternative antioxidant strategies and packaging systems, will further support the development of commercially viable, clean-label, and nutritionally enhanced meat products. Furthermore, the inclusion of inulin as a dietary fiber should be interpreted with caution, as it may not be suitable for all consumers. Investigating alternative fiber sources with better gastrointestinal tolerance is recommended in future reformulation efforts.

## Figures and Tables

**Table 1:** Bologna-type sausage formulation with varying levels of emulsion gel.

No.	Ingredient	Control (%)	7% EG	10% EG
1	Horse meat, grade 1	30.0	30.0	30.0
2	Beef, grade 1	37.0	30.0	27.0
3	Chicken meat	30.0	30.0	30.0
4	Emulsion gel	0.0	7.0	10.0
5	Modified starch	3.0	3.0	3.0
	Total raw materials	100.0	100.0	100.0
6	Table salt	1.0	1.0	1.0
7	Sodium nitrite	0.008	0.008	0.008
8	BIFCOLOR food coloring	0.7	0.7	0.7
9	Nitrite salt	1.0	1.0	1.0
10	BIOPRO 50 multifunctional mix	0.13	0.13	0.13

**Table 2:** Color values (L*, a*, b*), pH, and total fluid release of EG at 0 and 7 days of storage at 4 °C.

Parameter	Day 0	Day 7
L* (lightness)	71.45 ± 3.12 ^a^	78.90 ± 1.85 ^b^
a* (redness)	1.60 ± 0.18 ^b^	0.22 ± 0.06 ^a^
b* (yellowness)	20.95 ± 1.01 ^b^	16.55 ± 0.31 ^a^
pH	5.28 ± 0.05 ^a^	5.45 ± 0.06 ^b^
Total fluid release (%)	1.06 ± 0.06 ^a^	1.38 ± 0.09 ^b^

Values with different superscript letters in the same row indicate significant differences (*p* < 0.05).

**Table 3:** Proximate composition and energy values of sausages with different levels of EG incorporation.

Parameter	Control	7% EG	10% EG
Moisture (%)	67.82 ± 0.34 ^a^	68.95 ± 0.28 ^a^	69.63 ± 0.31 ^b^
Protein (%)	12.02 ± 0.41 ^a^	12.38 ± 0.36 ^b^	13.12 ± 0.29 ^c^
Fat (%)	12.21 ± 0.26 ^c^	11.48 ± 0.33 ^b^	10.57 ± 0.22 ^a^
Ash (%)	2.29 ± 0.08 ^a^	2.14 ± 0.07 ^a^	2.37 ± 0.05 ^b^
pH	6.12 ± 0.02 ^c^	5.56 ± 0.01 ^b^	5.43 ± 0.01 ^a^
Energy (kcal/100 g)	180.61 ± 1.85 ^c^	173.04 ± 2.01 ^b^	164.85 ± 1.67 ^a^
Energy from fat (kcal)	109.89 ± 1.76 ^c^	103.32 ± 1.43 ^b^	95.13 ± 1.38 ^a^

Superscript letters within the same row that differ indicate statistically significant differences (*p* < 0.05).

**Table 4:** Technological properties of sausage batters with varying levels of EG.

Parameter	Control	7% EG	10% EG
WHC (%)	83.5 ± 1.2 ^a^	86.9 ± 1.0 ^b^	89.8 ± 0.9 ^c^
TEF (%)	6.2 ± 0.3 ^c^	4.7 ± 0.2 ^b^	3.5 ± 0.2 ^a^
EFAT (%)	0.96 ± 0.05 ^c^	0.68 ± 0.04 ^b^	0.41 ± 0.03 ^a^
JFS (%)	6.5 ± 0.6 ^c^	3.8 ± 0.4 ^b^	2.1 ± 0.3 ^a^
PY (%)	91.2 ± 0.7 ^a^	94.3 ± 0.6 ^b^	96.5 ± 0.5 ^c^

Superscript letters within the same row that differ indicate statistically significant differences (*p* < 0.05).

**Table 5:** Fatty acid composition (g/100 g lipid), total cholesterol content (mg/100 g), and nutritional ratios of the sausages.

Component	Control	7% EG	10% EG
Myristic acid (C14:0)	3.10 ± 0.08 ^a^	1.89 ± 0.05 ^b^	0.83 ± 0.02 ^c^
Palmitic acid (C16:0)	26.85 ± 0.19 ^a^	20.45 ± 0.15 ^b^	11.10 ± 0.09 ^c^
Stearic acid (C18:0)	27.00 ± 0.20 ^a^	14.35 ± 0.18 ^b^	8.82 ± 0.06 ^c^
ΣSFA	56.95 ± 0.40 ^a^	36.69 ± 0.36 ^b^	20.75 ± 0.21 ^c^
Oleic acid (C18:1)	27.90 ± 0.06 ^a^	35.22 ± 0.08 ^b^	42.31 ± 0.09 ^c^
ΣMUFA	30.20 ± 0.10 ^a^	37.45 ± 0.14 ^b^	45.14 ± 0.15 ^c^
Linoleic acid (C18:2, Σn-6)	4.35 ± 0.08 ^a^	12.12 ± 0.12 ^b^	21.11 ± 0.17 ^c^
Linolenic acid (C18:3, Σn-3)	0.00 ± 0.00 ^a^	3.95 ± 0.05 ^b^	5.68 ± 0.03 ^c^
ΣPUFA	4.35 ± 0.10 ^a^	16.07 ± 0.11 ^b^	26.79 ± 0.16 ^c^
Total cholesterol (mg/100 g)	62.30 ± 0.26 ^a^	43.70 ± 0.23 ^b^	28.40 ± 0.22 ^c^
ΣPUFA/ΣSFA	0.08 ± 0.01 ^a^	0.44 ± 0.02 ^b^	1.29 ± 0.03 ^c^
n-6/n-3	–	3.07 ± 0.04 ^b^	3.72 ± 0.06 ^c^
IA	1.14 ± 0.01 ^a^	0.52 ± 0.02 ^b^	0.20 ± 0.01 ^c^
IT	3.30 ± 0.01 ^a^	1.04 ± 0.02 ^b^	0.41 ± 0.01 ^c^

SFA: saturated fatty acids, MUFA: mono-unsaturated fatty acids, PUFA: poly-unsaturated fatty acids, IA: index of atherogenicity, IT: index of thrombogenicity. ^a–c^ Superscript letters within the same row that differ indicate statistically significant differences (*p* < 0.05). Data are expressed as mean ± standard error of the mean.

**Table 6:** Instrumental quality parameters of the reformulated sausages.

Parameters	Control	7% EG	10% EG
L*	64.80 ± 0.35 ^b^	67.30 ± 0.17 ^a^	67.85 ± 0.25 ^a^
a*	14.85 ± 0.14 ^b^	13.10 ± 0.46 ^c^	13.15 ± 0.11 ^c^
b*	11.80 ± 0.12 ^bc^	12.70 ± 0.15 ^b^	14.10 ± 0.14 ^a^
Hardness (N)	8.00 ± 0.45 ^b^	9.65 ± 0.62 ^a^	9.35 ± 0.48 ^a^
Cohesiveness	0.54 ± 0.02 ^a^	0.43 ± 0.02 ^b^	0.35 ± 0.02 ^c^
Springiness (mm)	0.83 ± 0.01 ^a^	0.82 ± 0.01 ^a^	0.81 ± 0.01 ^a^
Gumminess (N)	4.40 ± 0.30 ^a^	4.25 ± 0.50 ^a^	3.30 ± 0.05 ^b^
Chewiness (N × mm)	3.70 ± 0.25 ^a^	3.50 ± 0.10 ^a^	2.75 ± 0.12 ^b^

^a–c^ Different superscript letters in the same row indicate significant differences (*p* < 0.05). Data are expressed as mean ± standard error of the mean.

**Table 7:** Sensory characteristics and overall acceptance ratings of control and modified Bologna sausages.

Parameters	Control	7% EG	10% EG
Color	8.58 ± 0.51 ^a^	8.55 ± 0.63 ^a^	8.56 ± 0.50 ^a^
Taste	8.17 ± 0.72 ^a^	8.25 ± 0.55 ^a^	8.35 ± 0.48 ^a^
Flavor	8.03 ± 0.78 ^a^	8.05 ± 0.68 ^a^	8.42 ± 0.60 ^b^
Texture	8.33 ± 0.78 ^a^	8.33 ± 0.64 ^a^	8.45 ± 0.53 ^a^
Overall acceptability	8.38 ± 0.65 ^a^	8.40 ± 0.70 ^a^	8.48 ± 0.56 ^a^

The treatments were formulated as follows: Control—standard formulation; 7% EG—7% emulsion gel; 10% EG—10% emulsion gel. ^a,b^ Different superscript letters in the same row indicate no significant difference (*p* < 0.05). Data are expressed as mean ± standard error of the mean.

## Data Availability

The raw data supporting the conclusions of this article will be made available by the authors on request (Almagul Nurgazezova, almagul.nurgazezova@hotmail.com).

## Supplementary Materials

The following supporting information can be downloaded at: https://www.mdpi.com/article/10.3390/foods14152553/s1, Table S1: Fatty acid composition of emulsion gel.
