# Supplementary material for: Nutritional and Technological Benefits of Pine Nut Oil Emulsion Gel in Processed Meat Products
Source: Foods. 2025 Jul 22;14(15):2553. doi: 10.3390/foods14152553 (PMC12345647; doi:10.3390/foods14152553)
Supplement: Supplementary file 1 [file foods-14-02553-s001.zip › foods-3748802-supplementary.pdf]

Table S1. Fatty acid composition of emulsion gel

Fatty Acid	Abbreviation	Content (%)
Palmitic acid	C16:0	6.81
Stearic acid	C18:0	2.80
Oleic acid	C18:1	17.30
Linoleic acid	C18:2	70.19
Linolenic acid	C18:3	0.79
Arachidic acid	C20:0	0.24
Gondoic acid	C20:1	0.68
Behenic acid	C22:0	0.21
Others	-	0.98
SFA (Total Saturated Fatty Acids)	-	10.85
MUFA (Monounsaturated Fatty Acids)	-	17.98
PUFA (Polyunsaturated Fatty Acids)	-	71.17
